# Effect of extrusion cooking on the chemical and nutritional properties of instant flours: a review

**DOI:** 10.12688/f1000research.140748.1

**Published:** 2023-10-18

**Authors:** Remigio Yamid Pismag, María Paula Polo, José Luis Hoyos, Jesús Eduardo Bravo, Diego Fernando Roa

**Affiliations:** 1 Faculty of Agricultural Science, Universidad del Cauca, Comuna 1, Cauca, Colombia

**Keywords:** precooking, instant powders, instant preparation, instant consumption

## Abstract

Satisfying the nutritional requirements of consumers has made food industries focus on the development of safe, innocuous, easy-to-prepare products with high nutritional quality through efficient processing technologies. Extrusion cooking has emerged as a prominent technology associated with the nutritional and functional attributes of food products. This review aims to establish a theoretical framework concerning the influence of extrusion parameters on the functional and nutritional properties of precooked or instant flours, both as end-products and ingredients. It highlights the pivotal role of process parameters within the extruder, including temperature, screw speed, and raw materials moisture content, among others, and elucidates their correlation with the modifications observed in the structural composition of these materials. Such modifications subsequently induce notable changes in the ultimate characteristics of the food product. Detailed insights into these transformations are provided within the subsequent sections, emphasizing their associations with critical phenomena such as nutrient availability, starch gelatinization, protein denaturation, enhanced *in vitro* digestibility, reduction in the content of antinutritional factors (ANFs), and the occurrence of Maillard reactions during specific processing stages. Drawing upon insights from available literature, it is concluded that these effects represent key attributes intertwined with the nutritional properties of the end-product during the production of instant flours.

## Introduction

The market for precooked flours has witnessed significant growth both in developed and developing countries, providing opportunities for industries engaged in offering these products ( Allied, 2020). Developing nations, particularly, seek viable alternatives that facilitate the processing of region-specific raw materials using cost-effective and highly efficient techniques. The aim is to enable the development of instant preparation foods that effectively cater to the nutritional needs of the population ( Ali, Singh, & Sharma, 2016; Mouquet, Salvignol, Van Hoan, Monvois, & Trèche, 2003; Onyeoziri, Torres, Hamaker, Taylor, & Kock, 2021; Sebio & Chang, 2000). Currently, consumers are increasingly aware of their food consumption, and the ingredients employed in their formulation. The response of the food industry has been offering secure and nutritionally superior products while employing processing technologies that ensure efficiency and productivity ( Akande *et al.,* 2017; Sandrin *et al.,* 2018). In this context, instant flours have gained prominence as indispensable products or ingredients that contribute to the development of foods with versatile nutritional, functional, and textural properties. Instant flours, often referred to as pregelatinized flours, undergo hydro-thermal modification processes to attain starch gelatinization in the raw materials ( Sebio & Chang, 2000). Pregelatinized flours primarily are made of starch-rich raw materials, such as cereals, tubers, and selected fruits. However, it is vital to acknowledge that plant-based flours encompass additional constituents besides starch, including proteins, fibers, lipids, and bioactive compounds ( Akande *et al.,* 2017; Espinosa *et al.*, 2021). Hence, the presence of these constituents must be considered during their processing, due to their influence on the physical, chemical, nutritional, and functional properties of the processed flours ( Transparency, 2019).

## Extrusion parameters to produce instant flours

Extrusion is a widely used technique in the creation of instant preparation foods such as beverages, porridges, infant foods, and desserts. Additionally, the raw materials employed in this process encompass a wide range, including cereal flours, legumes, fruits, tubers, and customized blends (see Table 1). The common raw materials used for producing precooked flours are rice, wheat, and maize. They have priority due to their wide availability and ease of being processed on an industrial scale. ( Transparency, 2019). Extrusion induces starch modification through the process of gelatinization ( Heredia *et al.,* 2019). However, it’s common to employ additional processes such as germination and enzymatic hydrolysis for starch modifications ( Tovar *et al.,* 2017; Pismag *et al.,* 2023). These supplementary procedures contribute to the creation of distinct functional properties in instant preparation products, with the objective of achieving effortless solubility and optimal water retention ( Sandrin *et al.,* 2018; Espinosa *et al.,* 2021). However, ensuring solubility and suspension formation may not be enough, it is essential to consider the target audience when developing instant foods. In the pursuit of nutritional adequacy, the inclusion of legume-derived proteins or defatted raw materials is put forward by certain researchers ( Espinosa *et al.,* 2021). In some cases, animal-based proteins, such as dairy proteins, have also been explored to achieve balanced nutritional profiles ( Heredia *et al.,* 2019).

**Table 1.** Operating conditions for obtaining instant preparation flours.

Product	Ingredient	Extrusion conditions				Reference
		Temperature (°C)	Moisture (%)	Speed screw (rpm)	Die (mm)	
*Single screw extruder*						
Extruded flour	Whole quinoa flour and sprouted quinoa flour	(96-115-111 – 100)	20-30	-		( Tovar, *et al.*, 2017)
Instant porridge flour	Chia seed and cassava flour blend	(50-150-250)	10-15-20	100	10	( Otondi *et al.*, 2020)
Instant beverage extruded flour	Green banana flour	(20-30-40-50)	15	600, 800, 1000	4.5	( Giraldo *et al.*, 2019)
Instant porridge flour	Millet flour	120	30-32	700	6	( Onyeoziri *et al.*, 2021)
Instant beverage flour, porridge and desserts	Defatted sesame seeds	50-170		50-240		( Ruiz *et al.*, 2022)
Instant flour	QPM corn flour	(50-60-70) – (80-90-100)	28	30-80		( Reyes *et al.*, 2003)
*Twin screw extruder*						
Extruded flours	Quinoa flour	(80-120-80) – (80-140-80) – (80-160-80) – (80 – 180-80)	25	480		( Song *et al.*, 2020)
Infant food	Quinoa flour, millet flour	(190)	18	260		( Dong *et al.*, 2021)
Infant foods	Quinoa flour, amaranth flour and Andean potato flour	(45 – 175 – 180)	-	1500		( Jiménez *et al.*, 2020)
Instant flour	Whey protein concentrate and rice flour	120-180	17-23	225-375	4	( Heredia *et al.*, 2019)
Instant porridge flour	Amaranth, bean, and corn flours	(60-130-130) - (60-130-170)	14-20			( Akande *et al.*, 2017)
Instant infant-food flour	Corn and mung bean flour	(40-70-100-108) – (40-70-100-192)	12,6 – 19,4	349-601	1,5	( Ali *et al.*, 2016)
Instant porridge flour	Broken rice and cowpea flour	100-140	15-25			( Nahemiah *et al.*, 2016)
Instant flour	Amaranth grain	(90-110-120) – (130-150-160)	12-16	240 – 300	4	( Atukuri *et al.*, 2019)
Instant beverage flour	Chickpea flour modified with α-amylase and alcalase	150	22,5	580		( Silvestre *et al.*, 2021)
Instant infant-food flour	Soy-rice mixed flour	35-45-55-125-140-150-135-120	14		3,5	( Mayachiew *et al.*, 2015)
Cold-hot beverage instant flour	Chickpea, corn and sorghum flour	50-80-100-120-150	20-24	317		( Wang *et al.*, 2019)

Table 1 provides an overview of instant preparation food types obtained through extrusion. It outlines key process conditions that significantly influence product characteristics. Parameters less frequently discussed in the literature encompass extrusion temperature, material moisture content, and screw speed. Additionally, less extensively studied factors, including die opening, feed rate, and the composition ratios of raw material mixtures, warrant further investigation. Notably, both single-screw and twin-screw extruders are employed in processing these raw materials, showcasing the versatility, and expanded horizons of extrusion-based techniques for innovative food development.

## Chemical and nutritional properties

Extrusion cooking is a sequential and staged process that allows for progressive modification of the materials fed into the extruder. The raw materials used in the production of precooked or instant flours has a significant amount of starch (from cereal processing), high protein content (from legume processing) or it is also possible fiber content (mixed with cereal bran) ( Ali *et al.,* 2016; Akande *et al*., 2017; Kaur *et al*., 2019; Otondi *et al.,* 2020). Moreover, these raw materials exhibit a potential complementary component such as lipids, vitamins, minerals and micronutrients ( Ali *et al*., 2016).

Understanding the behavior of main components during extrusion cooking is essential to predict the characteristics of the final products. The extent of modification or structural changes in these components depends on the thermo-mechanical conditions regulated or adjusted through process parameters in the extrusion equipment ( Ali *et al*., 2016; Nahemiah *et al*., 2018). Consequently, the resulting characteristics of the products can vary significantly, leading to desirable attributes such as starch gelatinization, protein denaturation, increasing the *in vitro* digestion, enhanced nutrient availability (including sugars, polypeptides, phenolic compounds, and vitamins), increasing the soluble dietary fiber and reducing the level of antinutritional factors ( Chulaluck *et al.*, 2008; Otondi *et al.,* 2020). Conversely, some papers describe the occurrence of Maillard reactions promoting a reduction in the protein digestibility as same as decreasing the content of thermolabile compounds like vitamins and phenolic compounds ( Alam & Aslam, 2020; Offiah, Kontogiorgos, & Falade, 2019; Singh, Gamlath, & Wakeling, 2007). This section specifically focuses on elucidating the influence of process parameter modifications on the chemical and nutritional properties of instant preparation flours.

## Free sugars

Extrusion cooking promotes the formation of low molecules weight from the starches. The main phenomena is called gelatinization, which is a particular process in extrusion that occurs at low moisture contents (12–45%), and involves the starch granule swelling, the leaching of amylose fractions and low molecular weight polysaccharides is also promoted. However, as thermo-mechanical conditions become more aggressive, fusion, depolymerization, and dextrinization of starches are induced ( Dalbhagat, Mahato, & Mishra, 2019; Yadav, Dalbhagat, & Mishra, 2022). Both amylose and amylopectin are fully exposed to the mechanical effect of the screws, leading to the production of low molecular weight structures, including the generation of sugars ( Singh, Gamlath, & Wakeling, 2007).

Table 2 demonstrates that the production of free sugars can occur during the extrusion process. High-temperature conditions, elevated moisture contents, and increased screw speeds contribute to significant levels of free sugar production in flours. Mild or low extrusion conditions, characterized by low temperature profiles, low moisture content, and high screw speeds, do not induce structural changes in starches, as reported by ( Martínez, Calviño, Rosell, & Gómez, 2014b; Martínez, Rosell, & Gómez, 2014a). Conversely, reversing these conditions during extrusion facilitates structural modifications in starches, leading to increased availability of free sugars in the extruded products ( Cheftel, 1986). This is relevant in legume extrusion, where the structural modification of oligosaccharides, such as raffinose, results in the increased production of reducing and non-reducing sugars ( Chauhan & Bains, 1988; Pham & Del Rosario, 1984). Controlling sugar levels during the extrusion process is crucial for ensuring the nutritional and sensory quality of extruded products ( Yadav *et al.*, 2022). The production of sugars must be regulated to prevent adverse effects on protein digestibility during extrusion processes, unless fermentation is desired ( Singh *et al.*, 2007).

**Table 2.** Effect of extrusion parameters on free sugars.

Raw material	Extrusion conditions	Barrel temperature	Moisture	Screw speed	Reference
Wheat flour	Moisture 3,6-21,8%	+	+	-	( Martínez *et al.*, 2014a)
Rice flour	Moisture 17–30% Temperature 110–140°C	+	+		( Martínez *et al.*, 2014a)
Rice and legume flour	Temperature 60–95°C	+	+		( Chauhan & Bains, 1988)
White bean and green bean flour	Moisture 30-45% Temperature 93-132°C Screw speed 100-200 rpm	+	+	+	( Pham & Del Rosario, 1984)

(+) Positive effect of process parameter on product parameter, (-) Negative effect of process parameter on product parameter.

## Protein solubility

Protein is a representative fraction of cereal or legume flours, which suffer modification depending on the extrusion process conditions. During extrusion the hydrothermal and mechanical conditions cause denaturation and aggregation of proteins, leading to reduced solubility ( Silvestre, Espinosa, Heredia, & Pérez, 2020). The treatment of proteins at high temperatures and high moisture contents, unfold their quaternary structures and become part of the melted material. Subsequently, these modified proteins polymerize, cross-link, and reorient themselves, forming longer and fibrous structures. This mechanism is particularly noteworthy in obtaining textured proteins. Most of the extrusion processes reported in Table 3 demonstrate that protein solubility tends to decrease with increasing temperature. This is likely due to the thermal effect inside the extruder, which promotes cross-linking of shorter protein units ( Pelembe, Erasmus, & Taylor, 2002).

**Table 3.** Effect of extrusion parameters on soluble protein content.

Raw material	Extrusion conditions	Barrel temperature	Moisture	Screw speed	Reference
Soybean meal	Moisture 15 to 27% Temperature 110 to 150°C Screw speed 300 rpm	-	-		( Singh, 2021)
Chickpea flour	Moisture 15 to 22% Temperature 143 to 150°C Screw speed 450 to 700 rpm	+	-	+	( Silvestre *et al.*, 2020)
Canola flour	Moisture 24, 30, 36%		-		( Zhang *et al.*, 2017)
Sorghum and cowpea flour blend	Moisture 200 g/Kg Temperature 130 to 165°C Screw speed 200 rpm	+			( Pelembe *et al.*, 2002)
Lentil flour	Moisture 18% Temperature 135 to 175°C Screw speed 500 rpm	-			( Li & Lee, 2000)
Wheat flour	Moisture 30% Temperature 60 to 160°C Screw speed 200 rpm	-			( Li & Lee, 1997)

However, some authors report an increase in protein solubility associated with higher barrel temperatures ( Silvestre *et al.*, 2020; Zhang *et al.*, 2017). This may be attributed to the breakdown of protein aggregates at high temperatures ( Singh, 2021). Comparing the reports of Singh (2021) and Silvestre *et al.*, (2020) where the maximum temperature and moisture content are similar, the screw speed is the determining factor for increased nitrogen solubility. The mechanical effect of this parameter is rarely reported, but it may contribute to the breakdown of protein aggregates, thereby increasing protein solubility. According to Table 3, solubility losses are evident with increasing moisture content ( Zhang *et al.*, 2017).

According to Zhang *et al.* (2017), the molecular weight distribution of canola proteins undergoes redistribution after the extrusion process, resulting in a decrease of higher molecular weights and increased concentration in intermediate molecular weights due to the formation of protein conglomerates, similar findings were described by Pelembe *et al.*, (2002). Although a reduction in protein solubility is widely reported, Li and Lee (2000) describe the associations or conglomerates formed under high extrusion conditions (temperature, and moisture content) are weak molecular bindings composed of non-covalent or fragile bonds easily breakable. Protein solubility is an outstanding characteristic in the formulation of instant foods. Nonetheless, this techno-functional attribute can be influenced during the extrusion process ( Table 3). Hence, the regulation of processing temperature and moisture content is in the interest of regulating the solubility of these processed flours.

## Browning index

Browning index is the quantification of non-enzymatic browning reaction. This chemical reaction is developed when a reducing sugar interacts with amino groups in protein fractions, resulting in brown pigmentation in products exposed to high-temperature cooking processes such as extrusion ( Singh *et al.*, 2007). The quantification of non-enzymatic Maillard browning holds significant importance not only due to its desirable attributes in food manufacturing, resulting in appealing colors and aromas that attract consumer preferences ( Ruiz *et al.*, 2008). However, it becomes crucial to control this reaction as it negatively impacts protein digestibility and causes amino acid depletion from a nutritional perspective ( Osen, Toelstede, Eisner, & Schweiggert, 2015). The development of brown color during the extrusion process is partly associated with lysine depletion caused by interactions with simple sugars. Lysine is an essential amino acid in protein synthesis ( Iwe, Van Zuilichem, Stolp, & Ngoddy, 2004). While the evaluation of non-enzymatic browning relies on color parameters determined through the CIElab protocol, the predictions derived from this index demonstrate remarkable precision in assessing the behavior of Maillard reactions production ( Ruiz *et al.*, 2008).

Most of the cereal, legume, or tuber flours encompass readily available free sugars within their matrix, which increase during the extrusion process due to gelatinization and the mechanical impact of the screws within the extruder equipment. Among the sugars documented as susceptible to Maillard reactions, glucose > d-mannose > d-fructose are considered more reactive as fundamental units compared to their dimers. A similar pattern of reactivity is observed for proteins, which exhibit enhanced responsiveness in Maillard reactions when present as basic constituents, namely amino acids ( Singh *et al.*, 2007; Yadav *et al.*, 2022). Maillard reactions are intensified when the available protein content in the raw material is higher, this is particularly noticeable in legume flours that are ready for such reactions ( Yadav *et al.*, 2022).

Table 4 shows the effect of extrusion conditions on the generation of Maillard reactions. It is apparent that during the extrusion of flours to produce instant foods, intensified Maillard reactions occur with higher process temperatures and screw speeds. This can be attributed to the thermal and mechanical effects, which break down both starches and proteins, resulting in simpler components that promote Maillard reactions. As a result, there is an increase in brown color, quantified by the browning index, correlated with a decrease in luminosity (L*) and increasing the red (a*) and yellow (b*) colors ( Waramboi, Gidley, & Sopade, 2013; Yadav *et al.*, 2022). High screw speed promotes a mechanical shearing force on the melted material as it crosses the barrel in the extruder, developing the damage of polysaccharides (starch), which can contribute production of the Maillard reaction ( Iwe *et al.*, 2004; Yadav *et al.*, 2022). On the other hand, it becomes apparent that the browning index decreases as the moisture content increases during the extrusion process. Although moisture content starts the Maillard reaction, a higher level of moisture increases the flow of the melted material, thereby diminishing its residence time and controlling the formation of this reaction. Water acts as a plasticizer in the process reducing the viscosity of the melted material ( Camire, 1991).

**Table 4.** Effect of extrusion parameters on the browning index.

Raw material	Barrel temperature	Moisture	Screw speed	Reference
Sorghum and barley blend	+	-	+	( Koa *et al.*, 2017)
Barley varieties	+	-		( Sharma *et al.*, 2012)
Soybean and sweet potato			+	( Iwe *et al.*, 2004)
Sweet potato flour	+	-		( Waramboi *et al.*, 2013)

## *In vitro* starch digestion

Englyst, Kingman, and Cummings (1992) developed a colorimetric method to describe the three starches fractions in food matrices. Rapidly Digestible Starch (RDS), indicating starch that undergoes digestion within 20 minutes based on hydrolysis rates; Slowly Digestible Starch (SDS), denoting starch that is digested between 20 and 120 minutes; and Resistant Starch (RS), which can be analyzed in three fractions and corresponds to the starch fraction that remains undigested after 120 minutes. This method relies on controlled enzymatic assessment using pancreatin and amyloglucosidase. The evaluation of these starch fractions provides information into the *in vivo* starch digestion rate ( Smrckova *et al.*, 2014; Zhang *et al.*, 2019).

The evaluation of *in vitro* starch digestibility is one of the extensively studied process parameters in extruded foods. this method is capable to reveal the changes that occur in the starch fraction of raw and processed materials, as same as their potential nutritional effects. Moreover, the rate at which modified starches can be digested, the proportion of starches available for digestion, and indirectly their relationship with glycemic index (GI) associated to the blood sugar content. This can be critical when RDS levels are elevated as they could negatively affect consumer health ( Qi *et al.*, 2021; Smrckova *et al.*, 2014). The available information regarding *in vitro* starch digestion in extruded instant flours is limited. Based on Table 5, it is possible to assess the impact of modifying the parameter process during extrusion on the starch fractions in the raw materials. In a general, it can be established that the modification of process parameters such as temperature, moisture, and screw speed exhibit an inverse correlation with the quantification of the Rapidly Digestible Starch (RDS) fraction, in comparison to the Slowly Digestible Starch (SDS) and Resistant Starch (RS) fractions. Within the extrusion process, alterations take place in each starch fraction because of gelatinization and shear forces. Consequently, RDS starches tend to convert into dextrin and/or simple sugars, while the SDS and RS fractions exhibit a decline content and may be quantified as RDS due to those modifications.

**Table 5.** Effect of extrusion parameters on *in vitro* starch digestion.

Raw material	Barrel temperature			Moisture			Screw speed			Reference
	RDS	SDS	RS	RDS	SDS	RS	RDS	SDS	RS	
Buckwheat flour (Tartary)	+	-	-	-	+	+				( Zhang *et al.*, 2023)
Pea flour	+	-	-							( Qi *et al.*, 2021)
Rice flour	+	-	-							( Lai *et al.*, 2022)
Bean varieties	+	-	-	-	+	+	+	-	-	( Ai *et al.*, 2016)
High amylose corn flour				-	+	+				( Zhang *et al.*, 2016)

RDS = Rapidly Digestible Starch, SDS = Slowly Digestible Starch, RS = Resistant Starch.

The variability in the *in vitro* starch digestion depends on the severity of the applied extrusion conditions to the raw material. Increasing the extrusion temperature leads to a high RDS content, attributable to modifications in the SDS and RS fractions ( Zhang *et al.*, 2023). The extrusion temperature constitutes one of the principal factors responsible for the gelatinization of available starches, causing the loss of their crystalline structure and the fragmentation of amylose and amylopectin chains due to the thermo-mechanical impact of the screw. This facilitates amylase accessibility during digestion ( Lai *et al.*, 2022). Conversely, increasing moisture content during the extrusion process reduces the mean residence time of the material inside extruder, resulting in higher quantifications of SDS and RS than RDS content. These results agree with the reduction in the *in vitro* starch digestion ( Zhang *et al.*, 2023). Water acts as a plasticizer capable to modify the gelatinization temperature of starches ( Koa *et al.*, 2017). Screw speed represents the factor with higher influence on the mechanical and shear forces during the extrusion process. Heightened screw speed enhances the starch structure modification through mechanical and shear-induced depolymerization, which impact the *in vitro* starch digestibility ( Koa *et al.*, 2017). Table 5 demonstrates that higher screw speeds yield a greater amount of RDS while reducing the proportion of SDS and RS.

## *In vitro* protein digestion

*In vitro* protein digestion is an indirect assessment of protein nutritional quality ( Akande *et al.*, 2017; Omosebi *et al.*, 2018). Plant proteins generally exhibit lower digestibility compared to the animal-derived proteins due to their amino acid configuration and the presence of antinutritional factors ( Atukuri *et al.*, 2019). The digestibility of plant proteins can be enhanced through treatments such as extrusion cooking. The fitted process parameters during extrusion and their intensity effect in the extruder influence protein digestibility. These factors impart different characteristics to the protein fractions found in the raw materials. From a nutritional perspective, the protein digestion extent is related to their level of denaturation, wherein their quaternary structures lose order and transition into lower-order structures, promoting the intestinal proteolysis during digestion ( Qi *et al.*, 2021; Ruiz *et al.*, 2008). Based on the studies in Table 6, it can be observed that, in most cases, an increase in temperature correlates with enhanced *in vitro* protein digestibility. However, some reports showed a reduction in the *in vitro* protein digestion when the raw materials were exposed to extremely high temperature conditions during the extrusion process (see Table 6).

**Table 6.** Effect of extrusion parameters on *in vitro* protein digestibility.

Raw material	Barrel temperature	Moisture	Screw speed	Reference
Defatted Quinoa and Chia	+			( Sánchez *et al.*, 2022)
Sesame Flour	+			( Ruiz *et al.*, 2022)
Pea Flour	+			( Qi *et al.*, 2021)
Amaranth Flour	+	-	+	( Atukuri *et al.*, 2019)
Corn and Soy Protein	+	+	+	( Omosebi *et al.*, 2018
Amaranth-Based Mixture	-	-		( Akande *et al.*, 2017)
QPM flour	+		+	( Reyes *et al.*, 2003)

QPM = Quality Protein Maize.

Between the most reported reaction involving proteins during the extrusion process encompass Maillard reactions, protein crosslinking, racemization, degradation, and the generation of lysinoalanine ( Martínez *et al.*, 2014a; Nahemiah *et al.*, 2017; Omosebi *et al.*, 2018; Ruiz *et al.*, 2022; Silvestre *et al.*, 2021). The quantification of these reactions tends to increase when proteins undergo excessive cooking during extrusion with a reduction in the *in vitro* protein digestion ( Reyes *et al.*, 2003). Furthermore, it can be observed that an increase in moisture content generally leads to a reduction in protein digestibility. This is attributed to the low residence time effect, which hinders structural modifications of the proteins. Conversely, the high screw speed increases protein digestibility due to the mechanical impact exerted by the screws on the raw material. Considering that the extrusion process requires a direct exposure of raw materials to high temperatures and relatively reduced moisture levels, a substantial quantity of complexes resulting from the reaction between reducing sugars and free amino acids is generated, leading to the occurrence of Maillard reactions. Then it becomes an important condition that should be regulated. The Maillard reaction imparts costumer pleasing flavors and colors, but as a uncontrolled reaction imposes limitations on protein availability and digestibility ( Alam & Aslam, 2020; Atukuri *et al.*, 2019). In extreme conditions it’s possible the formation of compounds such as acrylamides, which pose health risks and are synthesized when process temperatures overpass the threshold or when exposure time is long, even under low moisture content conditions during the extrusion process ( Alam & Aslam, 2020). Furthermore, higher screw speed during extrusion generates high shear forces, thereby boosting protein denaturation within the raw material and enhancing enzymatic hydrolysis during digestion ( Atukuri *et al.*, 2019). The excessive thermo-mechanical effect made during the extrusion process can also give rise to protein crosslinking. In addition, the high moisture content during the extrusion process prompts protein swelling and softening, consequently facilitating their denaturation and fractioning due to the thermo-mechanical impact within the extruder ( Atukuri *et al.*, 2019).

A few factors affecting *in vitro* protein digestibility during the extrusion process have been discussed thus far. However, it is notable to acknowledge that in plant-based raw materials, there exist other interfering elements that can also influence protein digestibility ( Qi *et al.*, 2021; Ruiz *et al.*, 2008; Silvestre *et al.*, 2020; Pico *et al.*, 2019). The presence of polyphenols in plant matrices can directly interact with digestive enzymes (such as amylase, trypsin, chymotrypsin, lipases), diminishing protein functionality and, consequently, reducing nutrient digestibility ( Omosebi *et al.*, 2018). However, the extrusion process can significantly decrease the presence of these interfering compounds in protein digestion and decrease the molecular weight of proteins to facilitate their digestion.

## Total phenolic compounds

The quantification of total phenolic compounds in vegetable matrices include free and bounded fractions and during extrusion process is possible to modify the relationship between the quantity of both fractions. Furthermore, it has been possible to observe a high quantification of total phenolic compounds when the extrusion conditions effectively break down the cell walls of the matrix ( Pico, *et al.*, 2020; Pico *et al.*, 2019). The increase of the temperature and screw speed during extrusion promotes the thermo-mechanical movement of the melted material inside the extruder, facilitating the release, extraction, and quantification of bound phenolic compounds, which then become part of the free phenolic fraction. Some phenolic species released include hydroxybenzoic acids, flavonols, catechins, and low molecular weight proanthocyanidins. However, it is important to note that certain studies report an increase in total phenolic compounds, while others report a decrease in this parameter, suggesting that the response may depend on the specific characteristics of the raw material and the employed extrusion conditions (see Table 7).

**Table 7.** Effect of extrusion parameters on total phenolic compounds.

Raw material	Barrel temperature	Moisture	Screw speed	Reference
Sesame Cake flour	+		+	( Ruiz *et al.*, 2022)
Defatted Quinoa and Chia Flour	+			( Sánchez *et al.*, 2022)
Buckwheat	-	+		( Cheng *et al.*, 2020)
Red Quinoa	-			( Song *et al.*, 2020)
Amaranth, Bean, and Corn Flours	-	-		( Akande *et al.*, 2017)
Green Banana Flour		-		( Sarawong *et al.*, 2014)

On the other hand, it has been observed that increasing the moisture content during the extrusion process tends to reduce the quantification of total phenolic compounds ( Akande *et al.*, 2017; Sarawong *et al.*, 2014). Water acts as a plasticizer, preventing the structural breakdown of the cell walls in the matrices and making it difficult to quantify the fractions of bound phenolics ( Jan *et al.*, 2017; Sarawong *et al.*, 2014; Wang *et al.*, 2022). However, it has also been reported that an increase in moisture content enhance the preservation of released phenolic fractions, as low moisture contents during the extrusion process can cause structural disruption or alteration of phenolic compounds ( Cheng *et al.*, 2020). Nevertheless, it is imperative to optimize the operational parameters of the extrusion process to prevent excessive temperatures and/or shear conditions. Most of the phenolic compounds are sensitive to temperature, which can induce their structural modifications, leading to the loss of their functionality and potential quantification as functional constituents in foods ( Wang *et al.*, 2022).

## Antinutritional Factors (ANFs)

Plant-based raw materials used in food production possess small quantities of a distinctive chemical compound, which are synthesized primarily for the plant’s survival and multiple physiological functions ( Elizalde *et al.*, 2009). Among these group of compounds are phytic acid, lectins, protease inhibitors (trypsin and chymotrypsin), saponins, oligosaccharides (raffinose and stachyose), and phenolic compounds (see Table 8). Consuming these components in high concentrations can lead to digestive issues, intoxication, allergies, and, in some cases, damage to intestinal epithelial cells, all these compounds have been grouped as antinutritional factors (ANFs) ( Elizalde *et al.*, 2009). ANFs possess high activity and exhibit the capability of conformation complexes with molecules of nutritional importance. Trypsin inhibitors disrupt the enzymatic activity during protein hydrolysis, thereby impeding its absorption ( Thirunathan & Manickavasagan, 2019). Lectins, as glycoproteins, readily bind to red blood cell and intestinal epithelial cell walls, causing harm and an adverse effect in the intestinal microbiota ( Vidal *et al.,* 2022). Phytic acid hinders mineral absorption at the intestinal level by forming complexes involving phytate-mineral-protein, which exhibit low solubility ( Vega *et al.*, 2010). Certain phenolic compounds are regarded as ANFs due to their capacity to bind with proteins, reducing protein solubility and digestibility ( Vidal *et al.,* 2022). Additionally, they influence consumption and acceptance due to production of bitter tastes and dark coloration ( Akande *et al.*, 2017; Omosebi *et al.*, 2018; Vidal *et al.*, 2022). Oligosaccharides, such as raffinose and stachyose, are high molecular weight sugars commonly found in most legumes ( Thirunathan & Manickavasagan, 2019). These sugars are indigestible due to their structure and the presence of α-1-6 galactose linkages that resist intestinal hydrolysis. Legume grains generally contain higher proportions of these sugars, which, according to some studies, display reduced sensitivity to temperature effects ( Elizalde *et al.*, 2009). However, their behavior can be influenced by processing conditions. Saponins, classified as glycosides of steroids or triterpenoids, display distinct characteristics such as imparting a bitter taste to quinoa and soybean seeds and their ability to generate foams, which interfere with mineral absorption in the intestine ( Elizalde *et al.*, 2009; Gee *et al.*, 1993).

**Table 8.** Effect of extrusion parameters on ANF content.

Raw material	Product parameter/condition	Barrel temperature	Moisture	Screw speed	Reference
Lentil flour	Raffinose	-			( Ciudad *et al.*, 2020)
	Stachyose	+			
Bean flour	Refinase	-	-		( Thirunathan & Manickavasagan, 2019)
	Stachyose	+	-	+	
Amaranth, Bean, and Corn Flours	Phytic Acid	-	-		( Akande *et al.*, 2017)
Quinoa Flour	Saponins	-	-		( Kowalski *et al.*, 2016)
Corn, soybean, and African Treculia blend	Phytic acid		+	+	( Nwabueze, 2007)
	TI			+	
Peanut, Corn, and Soybean Flour	TI	-	-		( Plahar *et al.*, 2003)
Soybean and Sweet Potato Flour Blend	TI		+		( Iwe & Ngoddy, 2000)
Lentil Flour	Tannins	-	+	-	( Ummadi *et al.*, 1995)
Soybean	TI	-	+	-	( Petres & Czukor, 1989)

TI = Trypsin Inhibitors, ANFs = Antinutritional Factors.

Tannins, categorized as phenolic compounds and recognized as ANFs, are present in the structure of various plant-based raw materials. Their consumption interferes with protein and mineral digestion as tannins form complexes with carbonyl groups in dietary proteins or enzymes ( Duguma, Forsido, Belachew, & Hensel, 2021; Nikmaram *et al.*, 2017; Qi *et al.*, 2021).

The ANFs in plant matrices interfere on nutrient absorption. Therefore, thermal treatments like extrusion are employed to mitigate their effects and enhance the overall digestibility of nutrients in pre-cooked cereal and legume flours. Table 8 shows the behavior of oligosaccharides, such as raffinose and stachyose, and the influence of the processing conditions applied during extrusion. It has been observed that increasing the temperature leads to a decrease in raffinose content while increasing stachyose content ( Ciudad *et al.*, 2020; Thirunathan & Manickavasagan, 2019). The reduction in raffinose can be attributed to structural modifications, including the cleavage of 1-2 furanosidic bonds, which occur due to exposure to high temperatures. On the other hand, the increase in stachyose at higher temperatures and screw speeds is associated with the thermo-mechanical effect on the food matrix, which facilitates the release of bound oligosaccharides from other macromolecules and the disruption of cell walls, allowing their extraction as soluble components ( Cotacallapa *et al.*, 2021). Similarly, Table 8 demonstrates that high temperatures inactivate the content of phytic acid due to its thermosensitivity and the formation of insoluble complexes with other components ( Omosebi *et al.*, 2018; Batista *et al.*, 2010). According to Rathod and Annapure (2016), both temperature and moisture content during the extrusion process contribute to the structural modifications of phytates in the raw materials. These changes may involve the cleavage of covalent bonds between amino groups and the hydrolysis of peptide bonds in aspartic acid residues, leading to the inactivation of phytates.

Likewise, the content of saponins exhibits a decrease with increasing temperature and moisture content during the extrusion process (see Table 8). This behavior is attributed to the shear and mechanical energy during extrusion, which alters the original structure of saponins, resulting in smaller chemical fragments and a reduction in the pungent or astringent taste in quinoa-based products ( Kowalski *et al.*, 2016). In addition, Table 8 shows that the trypsin inhibitors tend to decrease under intense thermal and mechanical effects during extrusion, leading to molecular degradation ( Batista *et al.*, 2010; Ciudad *et al.*, 2020; Konstance *et al.*, 1998). However, the trypsin inhibitors content increases when the extrusion conditions are less aggressive. The only condition that modifies this behavior is an increase in moisture content. While some reports suggest classifying ANFs based on their temperature sensitivity, it has been observed that the most affected by thermal and mechanical effects are protease inhibitors (trypsin and chymotrypsin inhibitors), including phytic acid and lectins ( Akande *et al.*, 2017; Balandran *et al.*, 1998; Camire, 1991; Elizalde *et al.*, 2009). Tannins can develop complexes during the extrusion process under strong processing conditions. High temperatures and high screw speeds induce a thermal and shear effect that modifies the structure of these phenolic compounds. However, when the extrusion conditions are milder, achieved by increasing the moisture content, the preservation of tannins in the final product is higher ( Ummadi *et al.*, 1995).

## Conclusions

This review has demonstrated the usefulness of the extrusion cooking process in obtaining instant flours known as pregelatinized flours. The extrusion cooking process conditions such as temperature, moisture content of the material and screw speed, directly affect the behavior of the raw material components during the process. The most used raw materials to obtain pre-cooked flours have in their composition a significant amount of starch, protein, lipids, and others like fiber, micronutrients such as vitamins and minerals, all of them exposed to be modified by the thermo-mechanical conditions. These modifications influence the nutritional and functional characteristics of the final product.

The final results of the process will be associated with the modification of the operative extruder parameters, finding as results characteristics that become beneficial for the final product such as starch gelatinization, protein denaturation, increased *in vitro* digestibility, nutrient availability, increased soluble dietary fiber and reduction in the content of anti-nutritional factors (ANF), all of these characteristics are linked to the nutritional and functional properties of the instant flours. However, there is a lack of information that must be investigated regarding the already mentioned process conditions and other new ones, in the same way, to study their effect on the processed raw materials. There are even raw materials whose behavior is unknown, and which may be important in obtaining instant flour by extrusion.

It should be noted that the moisture content and the temperature of the process influence the solubility of the proteins and the gelatinization of the starch, which is fundamental in the development of instant preparation foods; therefore, it is necessary to modulate the moisture content and the process temperature to keep the solubility of these processed flours under control. On the other hand, the increase in temperature increases the digestibility of protein *in vitro* and by extrusion cooking it is possible to improve the digestibility of proteins of vegetable origin. In the same way, the phenolic content is modified by increasing in availability of thermostable bonded species due to cell wall broken of raw materials due to thermo-mechanical conditions during extrusion process.

As it has been established so far, the presence of ANFs in plant matrices represents an important limitation on the levels of nutrient absorption, it is for this reason that heat treatments such as extrusion reduce their effect and enhance the effective digestibility of available nutrients from precooked cereal and legume flours.

## Data Availability

No data are associated with this article.
