# Supplementary material for: Effect of extrusion cooking on the chemical and nutritional properties of instant flours: a review
Source: F1000Res. 2024 Jan 12;12:1356. Originally published 2023 Oct 18. [Version 2] doi: 10.12688/f1000research.140748.2 (PMC10905115; doi:10.12688/f1000research.140748.2)
Supplement: Peer review history

# Reviewer report: Reviewer response for version 2

**DOI:** 10.5256/f1000research.161234.r220806

**Published:** 2024-02-21

**Authors:** Plaza-Dorado José Luis

**Affiliations:** 1 Universidad del Valle, Cali,, Colombia

This article methodically analyzes the influence of extrusion parameters on the functional and nutritional characteristics of precooked or instant flours. It systematically explores how variables such as temperature, screw speed, and moisture content of raw materials are connected to alterations in the structural composition of these flours. By providing a detailed examination, the review clarifies the complex interactions these parameters have with the end-product, both as an independent food item and as a key ingredient, emphasizing the detailed and multifaceted aspects of the extrusion cooking process.

 Some comments on the text:

 The introduction effectively sets the context by discussing the significance of precooked flours in both developed and developing nations. It could be beneficial to briefly mention the specific challenges or needs that these flours address in developing countries, providing a clearer link to why their growth is particularly notable in these regions.

 In the sentence "The aim is to enable the development of instant preparation foods that effectively cater to the nutritional needs of the population," consider specifying who or what's aim it is for clarity.

 The mention of "distinct functional properties" is interesting, but providing a brief example of what these properties might be could add more depth to your explanation.

 the phrase "tween screw extrusion" should be corrected to "twin screw extrusion."

 There are some minor typographical errors, such as "Neverthless" which should be "Nevertheless".

 When discussing Maillard reactions, it would be beneficial to also mention any potential benefits, such as flavor development, alongside the drawbacks like reduced protein digestibility and nutrient loss.

 The sentence beginning with "The mechanical effect of this parameter is rarely reported..." could be clearer. Consider specifying which parameter you're referring to by saying "The mechanical effect of screw speed..."

 Pay attention to grammatical details to ensure readability. For instance, "which are synthesized primarily for the plant’s survival and multiple physiological functions" could be rephrased for clarity.

 Avoid redundancy and aim for conciseness without losing meaning. For example, "The raffinose and stachyose resist the intestinal hydrolysis because mammals do not have the enzyme galactosidase able to cleave the linkage α-1-6 galactose found in those galacto-oligosaccharides" could be shortened to "Mammals lack the enzyme galactosidase to hydrolyze raffinose and stachyose due to their α-1-6 galactose linkage."

 The conclusion does a good job summarizing the impact of extrusion conditions on the nutritional components of flours.

Is the review written in accessible language?

Yes

Are all factual statements correct and adequately supported by citations?

Yes

Are the conclusions drawn appropriate in the context of the current research literature?

Yes

Is the topic of the review discussed comprehensively in the context of the current literature?

Yes

Reviewer Expertise:

Bioprocesses and Biotechnology, Food Engineering.

I confirm that I have read this submission and believe that I have an appropriate level of expertise to confirm that it is of an acceptable scientific standard.

# Reviewer report: Reviewer response for version 2

**DOI:** 10.5256/f1000research.161234.r247408

**Published:** 2024-02-21

**Authors:** Martha Isabel Sanchez

**Affiliations:** 1 Faculty of Agronomic Engineering, University of Tolima, Ibague, Tolima, Colombia

Approved.

Is the review written in accessible language?

Yes

Are all factual statements correct and adequately supported by citations?

Yes

Are the conclusions drawn appropriate in the context of the current research literature?

Partly

Is the topic of the review discussed comprehensively in the context of the current literature?

Yes

Reviewer Expertise:

Food Engineering, Food Science and Technology

I confirm that I have read this submission and believe that I have an appropriate level of expertise to confirm that it is of an acceptable scientific standard.

# Reviewer report: Reviewer response for version 1

**DOI:** 10.5256/f1000research.154135.r220796

**Published:** 2024-02-08

**Authors:** Martha Isabel Sanchez

**Affiliations:** 1 Faculty of Agronomic Engineering, University of Tolima, Ibague, Tolima, Colombia

This review presents in a comprehensive and organized way the theoretical framework concerning the influence of extrusion parameters on the functional and nutritional properties of precooked or instant flours, both as end-products and ingredients. It highlights the pivotal role of process parameters within the extruder, including temperature, screw speed, and raw materials moisture content, among others, and elucidates their correlation with the modifications observed in the structural composition of these materials.

 Below are some recommendations from the text:

 1. I recommend including a figure of the extrusion process/equipment where parts and some operating conditions are mentioned, considering that throughout the manuscript, reference is made to process/equipment operating conditions.

 2. It is necessary to include an explanation in the footer of each Table indicating the information and the meaning of the symbols (+) and (-).

 3. In the section on Maillard reactions, it is necessary to mention in more detail the type of raw materials involved, what type of colors are desirable or undesirable in the final product, and their effect on consumer preference.

 4. Conclusion " *However, there is a lack of information that must be investigated regarding the already mentioned process conditions and other new ones, in the same way, to study their effect on the processed raw material" *does not take into account information previously mentioned in the text, is broad, and has no basis in the text to be included as a conclusion of the manuscript.

Is the review written in accessible language?

Yes

Are all factual statements correct and adequately supported by citations?

Yes

Are the conclusions drawn appropriate in the context of the current research literature?

Partly

Is the topic of the review discussed comprehensively in the context of the current literature?

Yes

Reviewer Expertise:

Food Engineering, Food Science and Technology

I confirm that I have read this submission and believe that I have an appropriate level of expertise to confirm that it is of an acceptable scientific standard.

# Reviewer report: Reviewer response for version 1

**DOI:** 10.5256/f1000research.154135.r220802

**Published:** 2023-11-15

**Authors:** Mercedes Pedrosa

**Affiliations:** 1 Food Technology Department, INIA-CSIC, Ctra. de La Coruña, Marbrid, Spain

I have reviewed the paper entitled effect of extrusion cooking on the chemical and nutritional properties of instant flours: a review.

 In general, the paper is of interest and it is well written. However, the discussion according to the literature has to be improved.

 I do not agree with the sentence on page 3 that affirms that the parameters of extrusion are less discussed in the literature. There are many papers that compare the effect of temperature, moisture, speed screw, etc. on different compounds. Please revise this fact and add the corresponding references.

 The main criticism is about the values of the parameters that the authors discuss. The authors include the different values of the parameters chose in the table 1; however, these values do not appear in the other tables nor in the text.

 For example, in table 2. The first line the authors write that barrel temperature, moisture and screw speed affect the free sugars of wheat flour. However, they only write in the table the moisture values.

 This problem can be observed in all the tables. Moreover, from table 4 to 8, the values do not appear.

 In page 8, the authors write that table 5 demonstrates that higher screw speeds yield a greater amount of RDS….. However, in the 5 and in the text they do not write the values of these parameters.

 Something similar can be observed in the text. For example, the authors write about an increase in the temperature or moisture produce an  XXX  effect. However, it is impossible to know which the range of this increase is, or they write about milder extrusion conditions, but they do not say which are the values of the different parameter necessaries to establish these mild conditions.

 On page 5, the authors say that extrusion produces free-sugars. Please add an example of these sugars.

 In page 9 when they write about ANF, please correct the name oligosaccharides by galacto-oligosaccharides or oligosaccharides of raffinose family...

 Table 6. Many of these papers studied more than an ANF, please include them in the table. The raffinose and stachyose resit the intestinal hydrolysis because mammals do not have the enzyme galactosidase. Please correct the sentence.

 Phytic acid is not very thermosensitibe compounds.

 Page 11, the authors say that extrusion produced an increase of trypsin inhibitors; however there are many papers that establish the opposite trend.

 The extrusion reduces some ANF and the smallest amount present in the extruded products can exert a positive health effect. Please add some sentence.

Is the review written in accessible language?

Yes

Are all factual statements correct and adequately supported by citations?

Yes

Are the conclusions drawn appropriate in the context of the current research literature?

Partly

Is the topic of the review discussed comprehensively in the context of the current literature?

Partly

Reviewer Expertise:

Food Technology,  extrusion, bioactive compounds.

I confirm that I have read this submission and believe that I have an appropriate level of expertise to confirm that it is of an acceptable scientific standard, however I have significant reservations, as outlined above.

# Response

**Published:** 2024-01-10

**Authors:** Remigio Pismag

**Affiliations:** Cauca, Universidad del Cauca, Comuna 4, Cauca, Colombia

I do not agree with the sentence on page 3 that affirms that the parameters of extrusion are less discussed in the literature. There are many papers that compare the effect of temperature, moisture, speed screw, etc. on different compounds. Please revise this fact and add the corresponding references.

 R/ Dear reviewer,

 In this paper, we are discussing about the reduced amount of information about the extrusion process in the develop of instant food products specifically. We carry out a searching focused on these topics: instant powders, instant porridge, instant beverages, and other same products all of them obtained by extrusion.

 However, we had clarified this issue on page 3 following your recommendation.

 The main criticism is about the values of the parameters that the authors discuss. The authors include the different values of the parameters chose in the table 1; however, these values do not appear in the other tables nor in the text.

 R/ We have adjusted the table with the information required. In addition, we had extended our discussion around the table 1.  

 For example, in table 2. The first line the authors write that barrel temperature, moisture and screw speed affect the free sugars of wheat flour. However, they only write in the table the moisture values.

 R/ We have adjusted the table with the information required.

 This problem can be observed in all the tables. Moreover, from table 4 to 8, the values do not appear.

 R/ We have adjusted the tables with the information required.

 In page 8, the authors write that table 5 demonstrates that higher screw speeds yield a greater amount of RDS….. However, in the 5 and in the text they do not write the values of these parameters.

 R/ We have adjusted the tables with the information required.

 Something similar can be observed in the text. For example, the authors write about an increase in the temperature or moisture produce an  XXX  effect. However, it is impossible to know which the range of this increase is, or they write about milder extrusion conditions, but they do not say which are the values of the different parameter necessaries to establish these mild conditions.

 R/ We have adjusted all the tables with the information required. We are following your recommendation.

 On page 5, the authors say that extrusion produces free-sugars. Please add an example of these sugars.

 R/ We describe the type of sugars generated during extrusion of cereal flours and we added examples of free sugars reported in extrusion of legume flours.

 In page 9 when they write about ANF, please correct the name oligosaccharides by galacto-oligosaccharides or oligosaccharides of raffinose family...

 R/ We are following your recommendations, and this issue was fitted.

 Table 6. Many of these papers studied more than an ANF, please include them in the table. The raffinose and stachyose resit the intestinal hydrolysis because mammals do not have the enzyme galactosidase. Please correct the sentence.

 R/ We added the ANFs available in the papers and all of them were included. On the other hand, we included the sentence adjusted in our discussion.

 Phytic acid is not very thermosensitibe compounds.

 R/ We are following your recommendations, and this issue was fitted.

 Page 11, the authors say that extrusion produced an increase of trypsin inhibitors; however there are many papers that establish the opposite trend.

 R/ We have fitted this issue, and we have explained the soft extrusion condition promotes the release and preservation of trypsin inhibitors.

 The extrusion reduces some ANF and the smallest amount present in the extruded products can exert a positive health effect. Please add some sentence.

 R/ We are following your recommendations, and we added a sentence.
